# Good Visual Outcome in Post Traumatic Exogenous Endophthalmitis Caused by Trichophyton Species

**DOI:** 10.7759/cureus.16071

**Published:** 2021-06-30

**Authors:** Prakash Supahiah, Nooramad Abbas Bin Ahmad, Wee Min Teh, Nor Fadzillah Bt Abd Jalil, Norshamsiah Md Din

**Affiliations:** 1 Department of Ophthalmology, Universiti Kebangsaan Malaysia Medical Centre, Kuala Lumpur, MYS; 2 Department of Ophthalmology, Hospital Melaka, Melaka, MYS; 3 Department of Ophthalmology, Hospital Selayang, Selangor, MYS

**Keywords:** exogenous endophthalmitis, trichophyton species, vitrectomy, lens aspiration, antifungal

## Abstract

Trichophyton species is a dermatophytic fungus commonly found in the skin, nails, hair, and other organic matters such as palm trees and soil. We report a rare case of a 23-year-old man who had a penetrating injury to the eye from the leaves of a palm tree and subsequently developed exogenous endophthalmitis. Culture from the vitreous tap revealed Trichophyton sp as the causative organism. Early vitrectomy and adequate intravitreal injection of amphotericin B resulted in good visual outcomes in an otherwise blinding condition. This is the first reported case of exogenous endophthalmitis secondary to Trichophyton species. Early diagnosis and prompt treatment may help improve visual outcomes.

## Introduction

Post-traumatic exogenous endophthalmitis is a devastating event, especially when it is related to contamination with organic matter. The incidence of post-traumatic endophthalmitis was reported to be as high as 16.5% [1]. A spectrum of microbes that enter the eye following ocular trauma can cause endophthalmitis depending on the geographical location, type of injury, living environment, and time from injury to wound repair [2]. Endophthalmitis from fungus is relatively rare and, it should be suspected in injuries involving organic material. The final visual outcome after fungal endophthalmitis is generally poor despite considerable progress in vitreoretinal surgery and intraocular antifungal injections.

Most cases of fungal endophthalmitis are caused by mold, like Fusarium and Aspergillus [3]. Trichophyton species is a dermatophytic fungus, commonly found in the skin, nail, hair, and other organic matters such as palm trees and soil [3]. Fungal endophthalmitis is typically indolent with a latent period between weeks to months compared to bacterial endophthalmitis, which usually presents within days. The inflammation tends to be more localized and often confined to the anterior chamber, pupillary space, or the anterior vitreous face [4]. Trichophyton infection may cause progressive keratolysis and perforation, scleral extension, and endophthalmitis, if not treated promptly.

There are several guidelines for treatment of fungal endophthalmitis, but the efficacy of currently available antifungal agents is limited with a relatively high medical treatment failure rate. The optimum therapy for exogenous fungal endophthalmitis is not well established, particularly in selection, route of administration, and dosage of antifungal agents [4]. Surgical intervention is required to decrease the fungal load, enhance antifungal penetration and efficacy, and increase chances of better visual outcome. Early combined medical and surgical intervention to minimize tissue damage may prevent further worsening of the infection and result in favorable outcome [5-7]. We report a rare case of exogenous endophthalmitis secondary to Trichophyton species following ocular trauma with good visual outcomes.

## Case presentation

A 23-year-old man who works in an oil palm plantation, presented with reduced vision, eye redness, and tearing for two days prior to presentation. He gave a history of accidental trauma to his right eye by a fallen frond of an oil palm tree. There were no other ocular or medical illnesses. On examination, visual acuity in his right eye was hand movement (HM), but there was no relative afferent pupillary defect (RAPD). There was a full-thickness corneal laceration wound measuring 4mm extending from 4 to 6 o'clock position at the paracentral area of the cornea with vitreous tracking to the wound. There was minimal hyphema in the anterior chamber with cells of 3+, and the pupil was irregular. Fundus view was hazy because of a vitreous hemorrhage.

**Figure 1:**
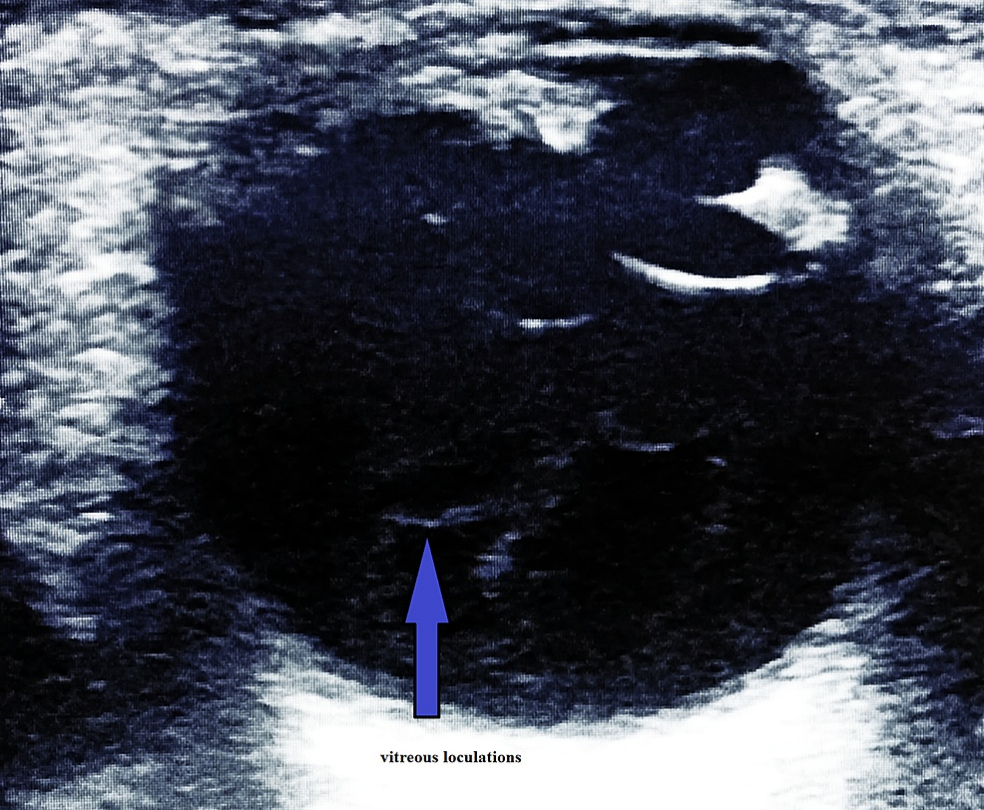
Post-operative B scan revealed vitreous loculations.

An orbital X-ray ruled out the presence of any radiopaque intraocular foreign body. He underwent toilet and suturing of the cornea followed by intravitreal tap and injection of vancomycin 2mg/0.1ml and ceftazidime 2mg/0.1ml. The vitreous tap was straw-colored. Postoperatively, the patient was started on topical Moxifloxacin 0.5% and Prednisolone acetate 0.12% every 2 hourly. He was also started on oral ciprofloxacin 500mg twice daily. Post-operative B scan revealed vitreous loculations (Figure 1). Trichophyton species were isolated from the vitreous culture. He was prescribed fluconazole 2% and amphotericin B 0.15% two hourly. In addition, oral fluconazole 200mg OD and intravitreal amphotericin 0.005mg was administered.

**Figure 2:**
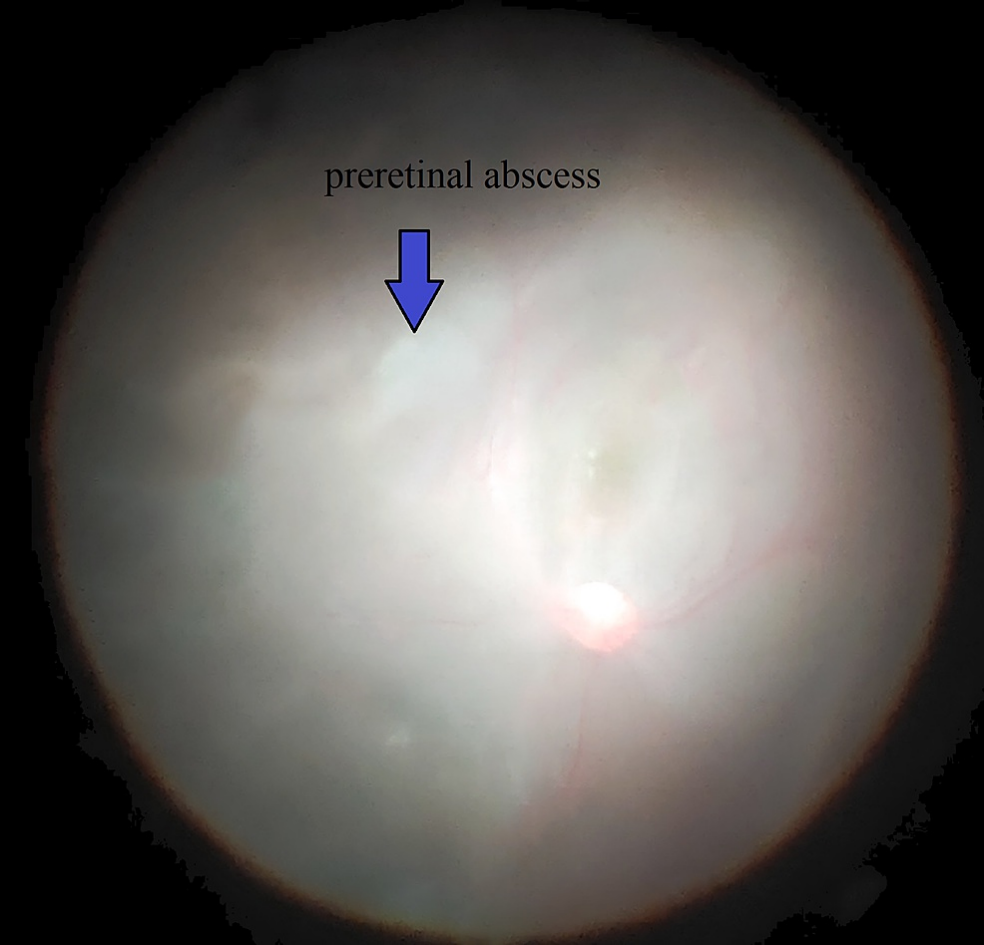
Preretinal abscess with patches of retinitis.

However, his symptoms worsened with increasing hypopyon and vitritis. We proceeded with pars plana vitrectomy and anterior chamber washout, followed by intravitreal amphotericin 0.05mg which was administrated. Intraoperatively, we observed a preretinal abscess with patches of retinitis (Figure 2). Eight days after the first vitrectomy, B-scan revealed retinal detachment. We proceeded with right eye lensectomy, vitrectomy, endolaser, and silicone oil tamponade. Intraoperatively, a small retinal hole was seen superiorly at 12 o'clock. Postoperatively, the patient's visual acuity in his right eye was 6/24 with a +10D lens. He was discharged well and was planned for secondary intraocular lens implantation with silicone oil removal at a later date. However, the patient defaulted the appointment and treatment.

## Discussion

Fungal endophthalmitis contributes to about 16.8% of the incidence of traumatic endophthalmitis. Literature shows that 85% of fungal endophthalmitis are caused by molds, particularly Fusarium and Aspergillus [8-12]. Trichophyton is a mold that has not been reported to cause traumatic endophthalmitis. It is present in almost all organic material and on the human body as a commensal [13]. Histologically, Trichophyton appears as smooth-walled macroconidia and microconidia. Macroconidia measures from 4-8 μm by 8-50 μm in size. They are mostly borne laterally directly on the hyphae or short pedicles, thin- or thick-walled, and club-shaped to fusiform. Microconidia is smaller, measuring 2-3 μm by 2-4 μm in size. They are spherical, pyriform to clavate, or of irregular shape, and they are few or absent in many species [14]. In general, most Trichophyton species can be isolated and identified on Sabouraud’s Dextrose Agar (SDA). Recently more specific molecular techniques have been used to differentiate between the four species. One such example is the T. mentagrophytes complex [15-16]. There are no specific clinical features present in Trichophyton sp to distinguish it from other fungal endophthalmitis species. However, Trichophyton sp has been reported to secrete a variety of proteases and collagenases which can lead to rapid progression of endophthalmitis and consequently loss of the entire eye [17].

There is no standard recommendation regarding the selection of antifungal regimes for Trichophyton-related endophthalmitis, although amphotericin B and fluconazole are the preferred antifungal agents in fungal endophthalmitis. In our patient, we chose a similar treatment regime for infections caused by other molds such as Aspergillus sp and Fusarium sp. Intravitreal injection of amphotericin B can achieve adequate therapeutic concentrations and reduce systemic toxicity [9]. Voriconazole is another antifungal drug that has efficacy against Trichophyton species, and some clinicians use voriconazole routinely as their first-line antifungal therapy [9-10]. Trichophyton species has been reported to grow in capsular bags leading to poor penetration of antifungals, necessitating the combination of lens aspiration, vitrectomy, and antifungal agents for fungal endophthalmitis [11]. Vitrectomy is important in the management of exogenous endophthalmitis to take fresh samples and decrease the load of microorganisms. In addition, the removal of vitreous gel may improve the diffusion of systemic antifungals [9-12]. With prompt identification and treatment, a good visual outcome was possible as achieved in our patient.

## Conclusions

A high index of suspicion for fungal-related traumatic endophthalmitis is pertinent as it allows for earlier and targeted treatment and protects against the development of serious inflammation and extensive damage to the eye. Early vitrectomy to reduce the organism load and facilitate penetration of antifungal drugs may improve the visual prognosis. Other measures such as repeated intravitreal injections and intensive topical antifungal treatment are essential to ensure the best possible outcome in these cases.
